# Refractory Graves’ Disease in an Adolescent Successfully Treated With Cholestyramine and Subsequent Thyroidectomy

**DOI:** 10.7759/cureus.66940

**Published:** 2024-08-15

**Authors:** Raghad Alsaidalani, Hesham Eltomy, Alaa B Aljuhani, Moutaz Osman

**Affiliations:** 1 Internal Medicine, King Fahad Military Medical Complex, Dhahran, SAU; 2 Surgery, King Fahad Military Medical Complex, Dhahran, SAU

**Keywords:** euthyroid, thyroidectomy., cholestyramine, adolescents, refractory graves

## Abstract

Graves’ disease is the most common form of hyperthyroidism in children and adolescents. There are three primary lines of treatment for Graves’ disease: antithyroid drugs (ATDs), thyroidectomy, and radioactive iodine. Ideally, patients should be rendered euthyroid before surgery to minimize complications.

Here, we report on a 14-year-old girl with severe Graves’ disease refractory to conventional treatments despite maximal therapy over 18 months. The patient received two types of ATDs, beta-blockers, and different courses of steroids; however, her thyroid function tests remained high. She was then given an adjunctive four-week course of cholestyramine, to which she responded well and became euthyroid. Subsequently, a thyroidectomy was performed without complications.

Cholestyramine is an effective adjunctive treatment for refractory Graves’ disease in adolescents.

## Introduction

Graves’ disease is the most common form of hyperthyroidism in children and adolescents [1]. It is an immune-mediated form of thyrotoxicosis, whereby the body forms autoantibodies, thyroid-stimulating hormone receptor antibodies (TRAB), that stimulate excessive production and secretion of thyroid hormone, as well as the growth of the thyroid gland. Clinically, this manifests as orbitopathy, dermopathy, diffuse goiter, and hyperthyroidism with its wide array of signs and symptoms [2]. The clinical manifestations of hyperthyroidism do not strongly correlate with the level of free thyroid hormones; rather, they correlate inversely with age, so the younger the patient, the more severe the manifestations [3,4].

The mainstay of treatment for Graves’ disease includes three options: radioactive iodine therapy (RAI), surgical removal of the thyroid gland, and antithyroid drugs (ATDs) such as propylthiouracil (PTU) and methimazole, or its prodrug carbimazole. Medical therapy usually precedes RAI or thyroidectomy to attain a euthyroid state prior to definitive therapy to prevent complications like worsening thyrotoxicosis or thyroid storm; this is usually attained in 1-3 months. The treatment regimen for Graves’ disease also includes beta-adrenergic blockade for patients with a heart rate of greater than 90 beats per minute, to both control the heart rate and peripherally block the conversion of free T4 to free T3 [5]. In the rare cases where a euthyroid state cannot be achieved by ATDs, the patient may be given potassium iodide and glucocorticoids. If the patient fails all previously mentioned lines of treatment, cholestyramine has been reported to successfully achieve a euthyroid state in a timely manner [6-8].

In this paper, we report on Graves’ disease in an adolescent refractory to conventional medical therapy, successfully treated with a short course of cholestyramine followed by total thyroidectomy [8,9].

## Case presentation

**Figure 1:**
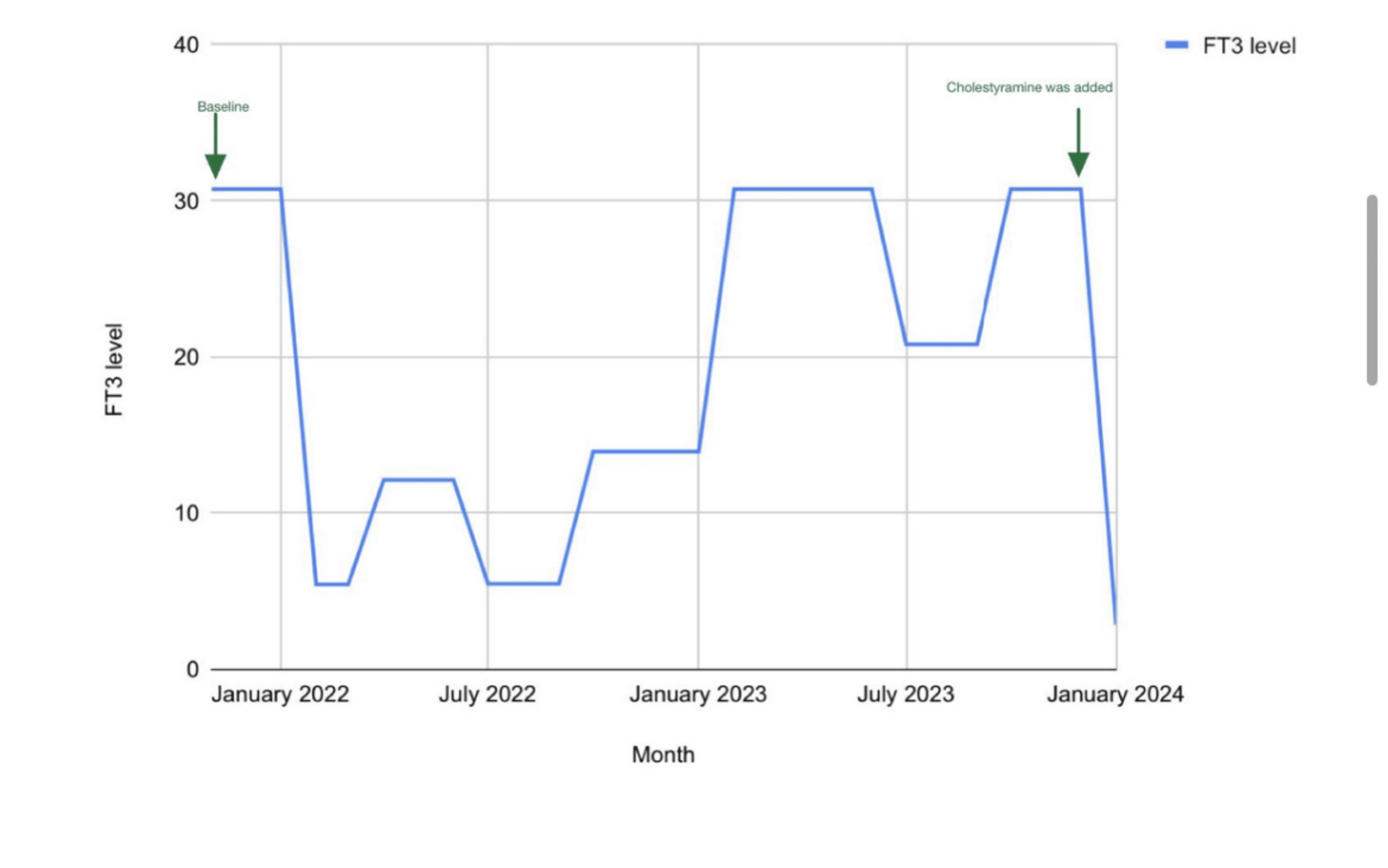
Trend of free triiodothyronine (FT3) throughout the course of treatment.

**Figure 2:**
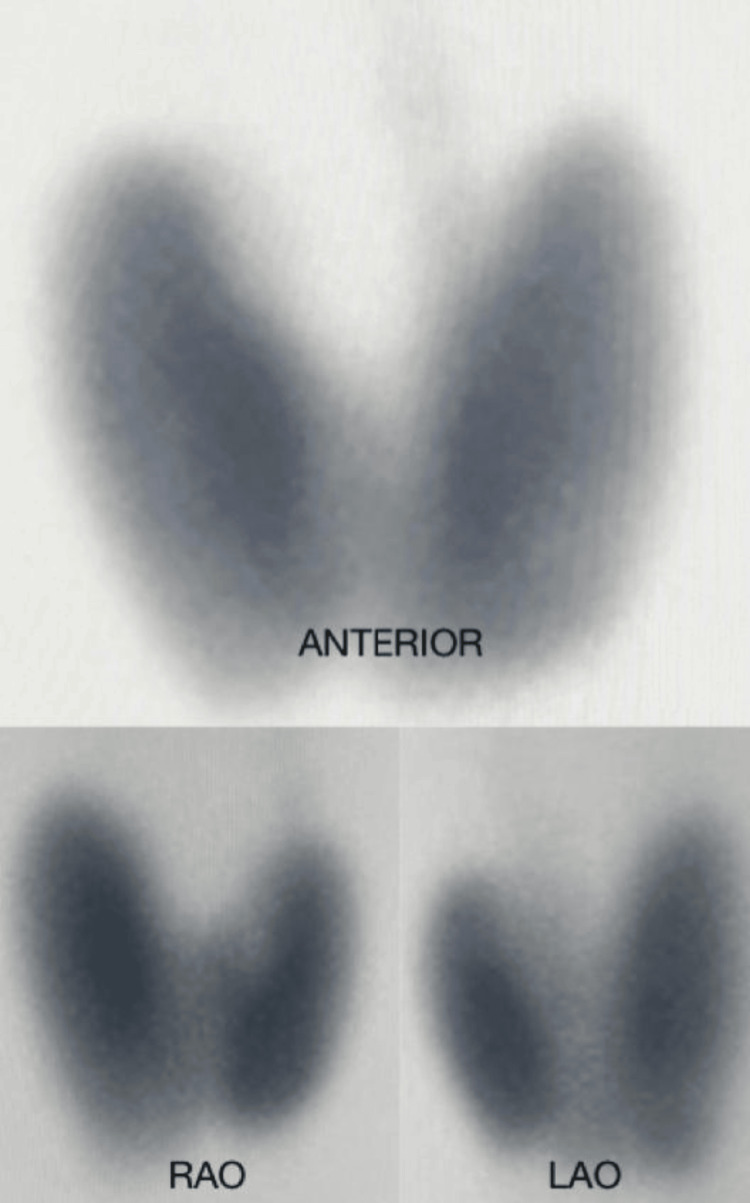
Thyroid scintigraphy (technetium-99m sodium pertechnetate) showing an enlarged thyroid gland with homogeneous, globally increased uptake, with no focal hot or cold nodules.

**Figure 3:**
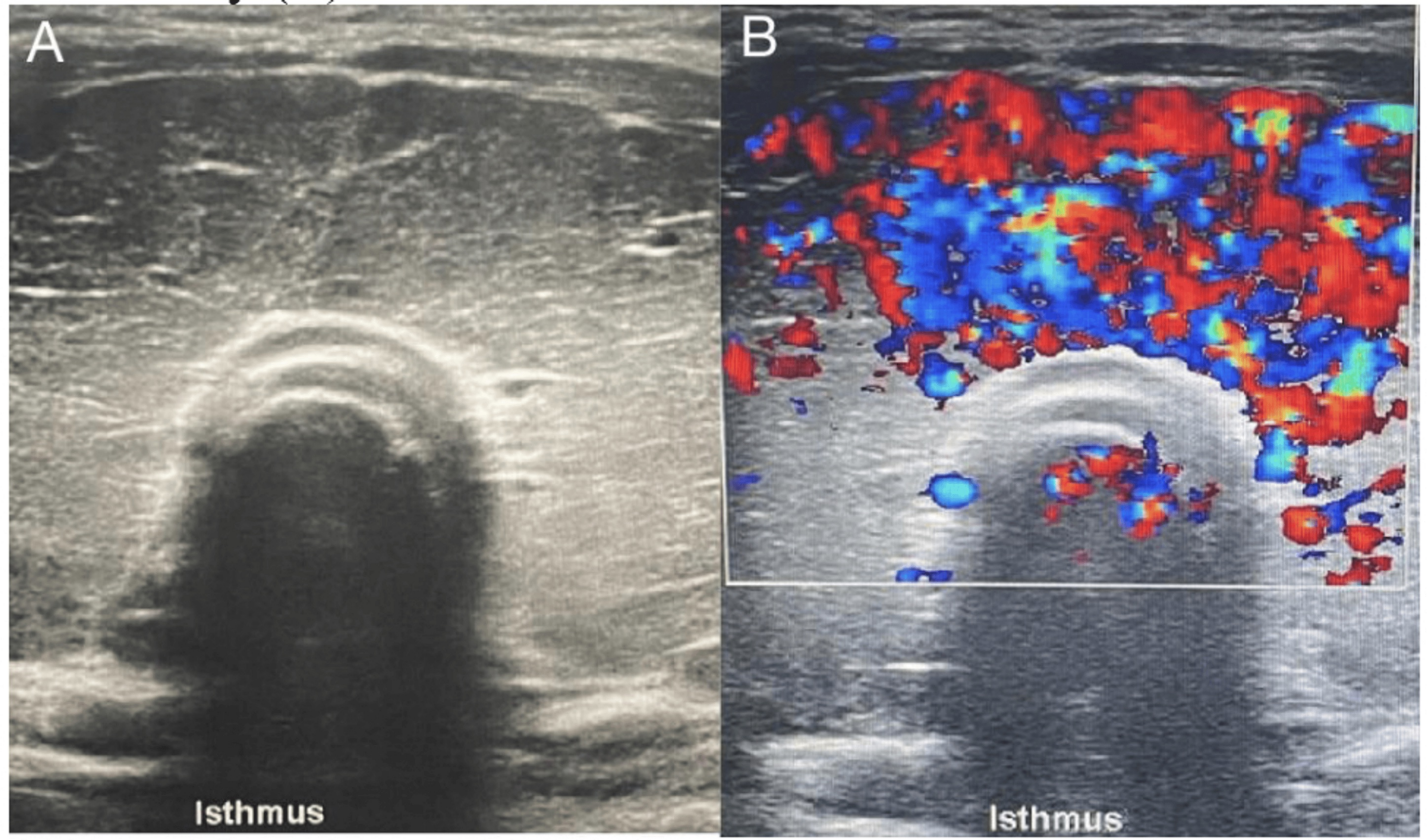
Thyroid ultrasound showing diffuse enlargement of both lobes of the thyroid gland with coarse, heterogeneous parenchymal echotexture (A). Color Doppler shows enhanced vascularity (B). N/A

A 14-year-old girl, not previously known to have any chronic illnesses, was referred from the primary health care unit of King Fahad Military Medical Complex, Dhahran, to the endocrine service in December 2023. The patient complained of excessive fatigue, eye protrusion, weight loss of about 4 kg over 3 months, irregular periods, and her mother noted a painless neck swelling. On examination, she had a blood pressure of 126/75 mmHg, a regular symmetrical pulse of 140 beats per minute, a normal respiratory rate, and oxygen saturation, and she was afebrile. She had bilateral fine tremors of the hands, mild bilateral exophthalmos, and a diffusely enlarged, firm, smooth, non-tender thyroid gland with a noted bruit. Her serum FT4 and FT3 levels were elevated, while her TSH was suppressed, and she had elevated levels of TSH receptor antibodies (Table 1 and Figure 1). The thyroid uptake and scan showed an enlarged gland with increased globally homogeneous uptake without focal hot or cold nodules (Figure 2). Meanwhile, an ultrasound of the thyroid gland revealed a diffusely enlarged thyroid gland, and color Doppler showed increased vascularity, all in keeping with Graves’ disease (Figure 3). Therefore, she was started on carbimazole 15 mg twice daily (BID) and propranolol 10 mg thrice daily (TID).

**Table 1:** Trend of free triiodothyronine (FT3), free thyroxine (FT4), thyroid-stimulating hormone (TSH), and TSH receptor antibody (TRAB) throughout the course of treatment, with relevant clinical remarks.

	Nov 2021	Feb 2022	Apr 2022	Jul 2022	Oct 2022	Feb 2023`	Jul 2023	Oct 2023	Dec 2023	Jan 2024
FT3 pmol/L Normal range (2.63-5.70)	>30.72	5.44	12.12	5.48	13.93	>30.72	20.79	>30.72	>30.72	2.86
FT4 Pmol/L Normal range (9-19)	52.27	<5.41	11.15	6.08	17.39	40.73	26.45	37.82	36.08	14.57
TSH MIU/L Normal range (0.35-4.94)	<0.0083	0.0103	<0.0083	0.7188	<0.0083	<0.0083	<0.0083	<0.0083	<0.0083	<0.0083
TRAB IU/L Normal range (<1.8)		>36.0								
Clinical remark	Baseline labs								Addition of cholestyramine	Admitted for thyroidectomy

The patient initially responded well to the therapeutic regimen during the first three months, and the dose of carbimazole was decreased to 10 mg BID. However, she relapsed and required increased doses of carbimazole 20 mg BID and propranolol 20 mg thrice daily; liver function tests continued to be normal. Compliance was emphasized at every visit, and definitive treatments were discussed with the family, radioactive iodine vs. surgery; the patient and her family agreed on surgery. The patient continued to have high FT3 and FT4, so carbimazole was increased to 20 mg TID, and prednisolone was also added. As the patient continued to be thyrotoxic, carbimazole was switched to PTU 200 mg three times daily. When the patient did not respond after two months of treatment, she was admitted to the hospital, given IV methylprednisolone for three days, and was discharged on dexamethasone 2 mg once daily, PTU 200 mg TID, propranolol 40 mg TID, and cholestyramine 4 grams TID was added. During her hospital admission, further emphasis on compliance was made, and it was encouraged for medications to be taken under family supervision.

A month later, her FT4 and FT3 completely normalized, and there was significant improvement in her symptoms. The patient was readmitted for a total thyroidectomy, which was performed uneventfully, and she was started on levothyroxine 100 mcg per day and tapering doses of prednisolone.

## Discussion

Graves’ disease has a wide range of manifestations, and the main determinant of the clinical signs and symptoms is age. The younger the patient, the more likely they are to exhibit the classical signs of hyperthyroidism, such as fine tremors of the hands, heat intolerance, increased perspiration, hyperphagia, polydipsia, hyperactive reflexes, nervousness, goiter, and sinus tachycardia rather than atrial fibrillation [10]. Our patient presented to the hospital complaining of neck swelling, symptoms of sympathetic overactivity, and protrusion of the eyes, which is the most common presentation of Graves’ disease in children and adolescents in Saudi Arabia [11].

Hyperthyroidism refractory to ATDs is a rare form of Graves’ disease that is potentially life-threatening. The underlying cause for this resistance to ATDs may be attributed to multiple factors, including but not limited to drug malabsorption, enhanced metabolism, the presence of antibodies against the drugs, decreased accumulation or aberrant action of the drugs in the thyroid gland, severity of the disease, especially severe elevation in FT3 levels, and finally, compliance [12]. These causes are important to consider while evaluating a patient with refractory Graves’ disease; however, it is notable that such extensive evaluations are not always feasible in the clinical setting.

Our patient initially responded partially to carbimazole 15 mg BID and propranolol 10 mg TID for a short period of three months. During the second visit, the carbimazole dose was decreased to 10 mg as a maintenance dose, and propranolol was discontinued as she had normal sinus rhythm. At the following appointment, she relapsed into hyperthyroidism. Initially, her relapse was attributed to poor compliance with the drug regimen. The importance of compliance was explained to both parents and the patient, and it was assured through pill counting and witnessed ingestion. Despite this, her thyroid function tests remained beyond the target range, and all the following medications failed to render her euthyroid: carbimazole, PTU, propranolol, prednisolone, methylprednisolone, and dexamethasone. It is important to note that inorganic iodide was not used in this patient as it was not available in our facility. Therefore, the most likely reason for her resistance to ATDs is the severity of the disease, especially the elevated levels of free triiodothyronine [13].

A few similar cases of refractory Graves’ disease have been reported worldwide in older patients who were successfully treated with cholestyramine and subsequent total thyroidectomy [6,7,14]. Cholestyramine is a bile acid sequestrant and an ion exchange resin most commonly used as a lipid-lowering agent, as it prevents the intestinal reabsorption of lipids. In the same manner, it prevents the reabsorption of thyroid hormones by binding to them in the intestinal lumen as they are excreted in the bile after being metabolized in the liver. The resin binds directly to conjugated and free thyroxine and free triiodothyronine, resulting in the interference of the enterohepatic circulation of the thyroid hormones and a subsequent decrease in their serum levels [8].

The earliest evidence of the use of cholestyramine in thyrotoxicosis comes from animal studies conducted in the 1960s by Bergman F et al. and Northcutt RC et al., where they found that the administration of cholestyramine in experimental animals, rats and hamsters, resulted in increased fecal excretion of radiolabeled thyroxine and decreased intestinal absorption of the hormone [15,16]. Then in the 1990s, Solomon BL et al. performed a double-blind, placebo-controlled, cross-over study evaluating the efficacy of cholestyramine as an adjunctive agent in the management of thyrotoxicosis in fifteen patients, mostly with Graves’ disease. They found that thyroid hormone levels rapidly declined in the first weeks of treatment with cholestyramine [8]. Similarly, Mercado M et al. performed a randomized control trial for one month comparing (I) methimazole 30 mg per day, 120 mg of propranolol per day, and cholestyramine 12 grams per day, all divided into three daily doses, (II) methimazole and propranolol alone, and (III) two weeks of methimazole, propranolol, and cholestyramine followed by two weeks of methimazole and propranolol alone. They found a greater decline in thyroid hormone levels in the first group compared to the second. The first two weeks of the third group were similar to the first group in terms of the rate of decline in thyroid hormone levels; however, in the subsequent two weeks, once cholestyramine was stopped, the rate of decline decreased and became similar to that of the second group. Importantly, they did not report any significant side effects during the treatment [9]. Tsai WC et al. showed comparable results in a randomized control trial using PTU instead of methimazole [17].

Although there is cumulative evidence on the efficacy of cholestyramine as an adjunctive treatment to ATDs in Graves’ disease, it remains infrequently used, likely because a euthyroid state is usually attained by using ATDs followed by RAI or thyroidectomy without the need to resort to other lines of treatment. However, in our patient, a euthyroid state could not be achieved by carbimazole, PTU, propranolol, and different courses of steroids. She also had Graves’ orbitopathy, so RAI could not be used. She responded well to a short course of four weeks of cholestyramine and subsequent thyroidectomy. The reported side effects of cholestyramine include belching, changes in bowel habits, flatulence, and abdominal discomfort, to name a few. However, the patient did not report any of these side effects during the course of her treatment [6,7,14].

## Conclusions

In conclusion, cholestyramine is a well-tolerated and effective adjunctive treatment for adolescents with Graves’ disease that is refractory to conventional lines of treatment. It is also a valuable preoperative drug for the rapid achievement of a euthyroid state in adolescents.
